# Biomechanical analysis of fibular graft techniques for nontraumatic osteonecrosis of the femoral head: a finite element analysis

**DOI:** 10.1186/s13018-020-01867-4

**Published:** 2020-08-17

**Authors:** Jian Xu, Shi Zhan, Ming Ling, Dajun Jiang, Hai Hu, Jiagen Sheng, Changqing Zhang

**Affiliations:** 1 Orthopedic Biomechanical Laboratory of Department of Orthopedic Surgery, Shanghai Jiao Tong University Affiliated Sixth People’s Hospital, No. 600, Yishan Rd, Shanghai, 200233 People’s Republic of China; 2 Department of Orthopedics, Fudan University Affiliated Huadong Hospital, Shanghai, 200040 People’s Republic of China; 3 Department of Orthopedic Surgery, Shanghai Jiaotong University Affiliated Sixth People’s Hospital, Shanghai, 200233 People’s Republic of China

**Keywords:** Osteonecrosis of the femoral head, Free vascularized fibula graft, Surgical techniques, Finite element analysis

## Abstract

**Background:**

Free vascularized fibula graft (FVFG) techniques have most consistently demonstrated beneficial effects in young patients diagnosed with nontraumatic osteonecrosis of the femoral head (NONFH), and the core track technique (CTT) in particular is the most commonly used technique. As an alternative to CTT, the modified light bulb technique (LBT) has been reported to have a higher success rate. However, its biomechanical outcomes are poorly understood. This study aimed to compare the biomechanical properties of modified LBT with those of CTT in treating NONFH.

**Methods:**

Two types (C1 and C2) of NONFH finite element models were established on the basis of a healthy subject and the Japanese Investigation Committee (JIC) classification system, and the CTT and LBT procedures were simulated in each type of model. The average von Mises stresses and stiffness of the proximal femur were calculated by applying a load of 250% of the body weight on the femoral head to simulate walking conditions. In addition, two patient-specific models were built and simulated under the same boundary conditions to further validate the LBT.

**Results:**

In the healthy subject-derived models, both the LBT and CTT resulted in reduced stresses in the weight-bearing area, central femoral head, femoral neck, and trochanteric and subtrochanteric regions and increased structural stiffness after surgery. In the weight-bearing area, the CTT reduced the stress more than the LBT did (36.19% vs 31.45%) for type C1 NONFH and less than the LBT did (23.63% vs 26.76%) for type C2 NONFH. In the patient-specific models, the stiffness and stresses also increased and decreased, respectively, from before to after surgery, which is consistent with the results of healthy subject-derived models.

**Conclusion:**

The biomechanical effects of the LBT and CTT differ by the JIC type of NONFH. In terms of preventing the collapse of the femoral head, the LBT may be more effective for JIC type C2 NONFH and may be a suitable alternative to the CTT, while for JIC type C1 NONFH, the CTT is still a better choice. Both techniques can improve the biomechanical properties of NONFH by reducing the proximal femoral stress and increasing the structural stiffness.

## Background

Nontraumatic osteonecrosis of the femoral head (NONFH) is a common disabling disease that mainly affects young individuals, and it is caused by insufficient blood supply and leads to femoral head collapse and premature osteoarthritis [1–3]. Various preservation procedures have been attempted to prevent the femoral head from collapsing. When the femoral head collapses, hip replacement is necessary, and young patients are reluctant to undergo this revision surgery due to the risks and financial burden [4, 5]. Among the preservation procedures, the free vascularized fibula graft (FVFG) procedure has most consistently demonstrated benefits in treating early NONFH, and it includes the removal of necrotic lesions under weight-bearing area, buttressing articular surface by grafted fibula, and the revascularization of the femoral head [2, 6–10].

The currently performed FVFG procedures mainly include the core tack technique (CTT) [11] and modified light bulb technique (LBT) [10], with success rates of 60–90% and 94.6–96%, respectively [2, 10–13]. The CTT is the most commonly used technique for treating NONFH, which is fulfilled by drilling a core tunnel from the lateral aspect of the greater trochanter, removing the necrotic bone tissue and implanting the bone. However, this technique requires a large amount of healthy bone to be reamed, longer fibula graft to be harvested, and a longer fibular pedicle, and the operation duration is long. An alternative to the CTT, the LBT, was initially described by Rosenwasser et al. [14] and subsequently modified by Zhang and his colleagues to be one of the FVFG techniques [10]. For the modified LBT, a window is created in the anterolateral cortex of the femoral neck. Anatomically, the lateral femoral circumflex vessel is constantly located in this anterior approach, which favourably allows smaller donor grafts and shorter fibular pedicles to be used [13]. In a long-term follow-up study, Gao et al. [10] reported that 91% (526/578) of the femoral heads remained in shape or even improved after LBT, according to the radiographic evaluation. Although the LBT is associated with a relatively higher success rate than is the CTT, Aldridge and Urbaniak [15] argued that the LBT can increase the stress by creating an anterior window. To the best of our knowledge, no quantitative study exists regarding the biomechanical benefits of the LBT, whose biomechanical outcomes have been poorly understood.

In this study, we aimed to investigate the biomechanical effects of LBT compared with those of the CTT in the treatment of NONFH. The hypothesis was that the clinical success rate is higher for the LBT partly because it has better biomechanical outcomes when it is used for the treatment of a certain type of NONFH.

## Methods

### Establishment of the initial 3D model

A healthy male volunteer who was aged 35 years, was 178 cm tall, and weighed 75 kg was recruited and had no history of hip trauma, hormone use, and or long-term alcohol consumption. The experiment was performed after the volunteer provided consent and the protocol was approved by the local ethics committee. His hip health was confirmed by an anteroposterior pelvis X-ray scan, a full-length X-ray scan of both lower extremities, and a thin-slice CT scan (SOMATOM Definition AS1; Siemens). A thin-slice CT scan of the left lower limb, from the pelvis to the feet, was taken with a resolution of 512 × 512 pixels and a layer thickness of 0.625 mm. With Mimics 19.0 (Materialise Ltd., Leuven, Belgium), segmentation techniques were used to reconstruct 3D models of the hip, femur, and fibula.

### Classification of the necrotic lesions of the femoral head

**Fig. 1:**
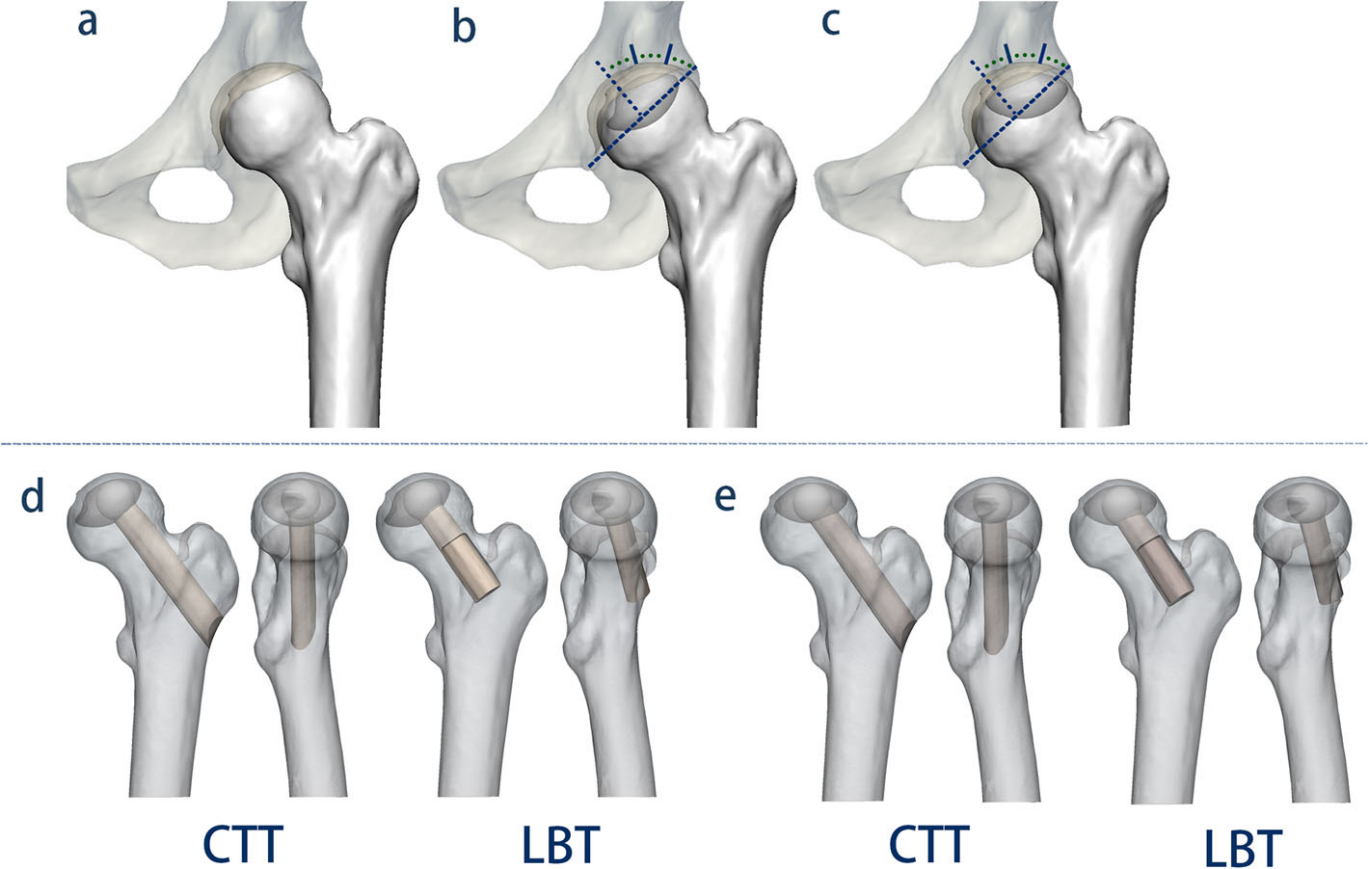
The healthy subject-derived models. **a** Reconstructed healthy hip model; **b** Japanese Investigation Committee (JIC) classification type C1: the lesions occupy more than medial two thirds of the weight-bearing portion but do not extend laterally to the acetabular edge. **c** JIC type C2: the lesions occupy more than medial two thirds of the weight-bearing portion and extend laterally to the acetabular edge. **d** The postoperative models on anteroposterior and medial-lateral views of the CTT and LBT in the JIC C1 group. **e** The postoperative models on anteroposterior and medial-lateral views of the CTT and LBT in the JIC C2 group

The necrotic lesions were classified into four visualized types (type A, B, C1, and C2), based on their location relative to the weight-bearing area according to the Japanese Investigation Committee (JIC) classification system [16]. In the JIC classification system, type C1 and C2 are recommended to undergo joint-preserving therapies. Thus, these types were included in this study and established based on the 3D finite element model of the normal upper femur (Fig. 1a–c). The criteria for the intervention for femoral head collapse was a necrotic domain volume of 30% of the femoral head [17].

### Simulation of the CTT and LBT

In the two necrotic groups, four postoperative models were simulated under the CTT and LBT surgical techniques (Fig. 1d, e). The models were simulated using Boolean operations as virtual surgical procedures in 3-Matic 11.0 (Materialise Ltd., Leuven, Belgium). For the CTT, a core along the axis of the femoral neck was drilled, starting from the lateral cortex at approximately 2 cm distal to the vastus ridge and ending at the necrotic lesion in the femoral head [11, 18]. The LBT opened a window at the anterior aspect of the femoral neck that matched the grafted fibula, with a size of 3 × 1.5 × 1.5 cm. A bone tunnel was made along the axis of the femoral neck from the window deep into the necrotic lesion in the femoral head [10, 13]. The debridement region removed with the burr was measured to be 1/2 of the radius of the necrotic domain [19]. The grafted fibular dimensions (length is shown in Table 1, and the fitting radius was 6.8 mm) were obtained from the volunteer’s CT images. The direction of the fibular axis was defined by the starting point, which was located in different starting places depending on the procedure, and the ending point, which was located in the central position of the weight-bearing area of the necrotic lesions in the anteroposterior and medial-lateral views. The distance between the cortical bone and the apical tip of the fibula was 5 mm [20]. The impaction cancellous bone was filled by the remaining voids.

**Table 1:** Length of grafted fibular and model elements

	JIC C1 Group			JIC C2 Group		
	Preoperation	CTT	LBT	Preoperation	CTT	LBT
Length*	-	9.43	6.53	-	9.53	6.27
Element	386995	461922	417666	384123	452905	435146
Node	81528	98634	89455	80980	97152	93317

*Length of grafted fibular (cm)

### Finite element analysis

All finite element analysis (FEA) models were created with a 1-mm mesh size in Abaqus/Standard 6.14 (SIMULIA Co., Providence, RI, USA). The number of elements and nodes for each FEA model is shown in Table 1. There was a linear correlation between bone density and the Hounsfield units. Bone density is related to the material properties. Hence, the material properties of each femoral model were based on the Hounsfield units from the CT scan data [21]. The mathematical formulas are as follows:

1 $$ \rho \left(\mathrm{g}/{\mathrm{cm}}^3\right)=0.000968\times \mathrm{HU}+0.5 $$

2 $$ \mathrm{If}\ \rho \le 1.2\mathrm{g}/{\mathrm{cm}}^3;E=2014{\rho}^{2.5}\left(\mathrm{Mpa}\right),\nu =0.2 $$

3 $$ \mathrm{If}\ \rho >1.2\mathrm{g}/{\mathrm{cm}}^3;E=1763{\rho}^{3.2}\left(\mathrm{Mpa}\right),\nu =0.32 $$

where *ρ* was the bone density, HU represented the Hounsfield units, *E* was the modulus of elasticity, and *ν* was Poisson’s ratio.

The fibula, necrotic bone, and cancellous bone were assigned to have different material properties, which were the same as those reported in other studies [19, 22, 23]. In these models, each part was assumed to be linear elastic, homogeneous, and isotropic. The moduli of elasticity were 15100 Mpa, 124.6 Mpa, and 445 Mpa, and the Poisson’s ratios were 0.3, 0.152, and 0.22, respectively. There were no gaps around the interfaces between the grafted bone and femur in any of the postoperative models.

**Fig. 2:**
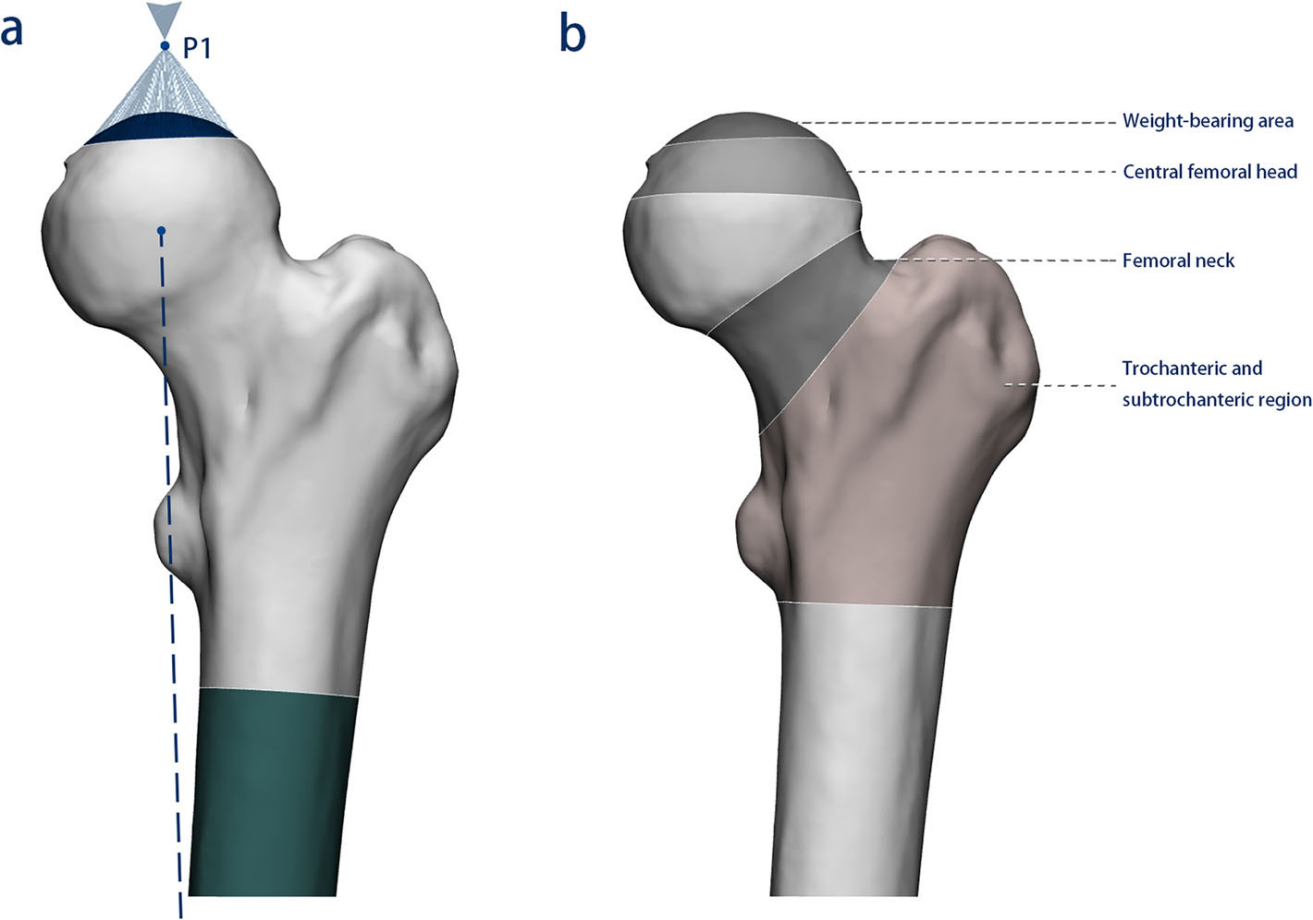
Boundary conditions and calculated regions in the proximal femur. **a** A load of 250% of the body weight was applied on the reference point (P1), which was coupled with the entire weight-bearing area along the mechanical axis (dotted line) of the femur. The distal femur was fully restrained to movement. **b** Four regions were calculated: the weight-bearing area, central femoral head, femoral neck, and trochanteric and subtrochanteric regions

To simulate a real situation for the hip joint, each FEA femoral model was fixed in the standing position. The same reference point on the weight-bearing area was set, and the elliptical area at the junction of the femoral head and the acetabulum was set to couple the entire weight-bearing area, in which the arc of the area towards the centre of the femoral head was 85° in the medial-lateral and anteroposterior views. A force of 250% of the body weight was loaded on the reference point along the mechanical axis of the femur to simulate the hip joint reaction force during normal walking [24], and the distal femur was fully restrained to movement (Fig. 2a).

In this study, for each model, the average von Mises stress in the mechanical conditions of the femur was calculated from all the elements in four different regions: (a) the weight-bearing area, (b) the central femoral head, (c) the femoral neck, and (d) the trochanteric and subtrochanteric region [25] (Fig. 2b). The structural stiffness was based on the ratio of the force to the displacement of the reference point, reflecting the ability of the proximal femur to resist deformation. The maximum principal strain at each element of the proximal femur was calculated to determine the risk of fracture in each region with respect to the ultimate compressive strain (0.0104) and ultimate tensile strain (0.0073) reported in previous studies [25, 26].

### Validation of the patient-specific models

**Fig. 3:**
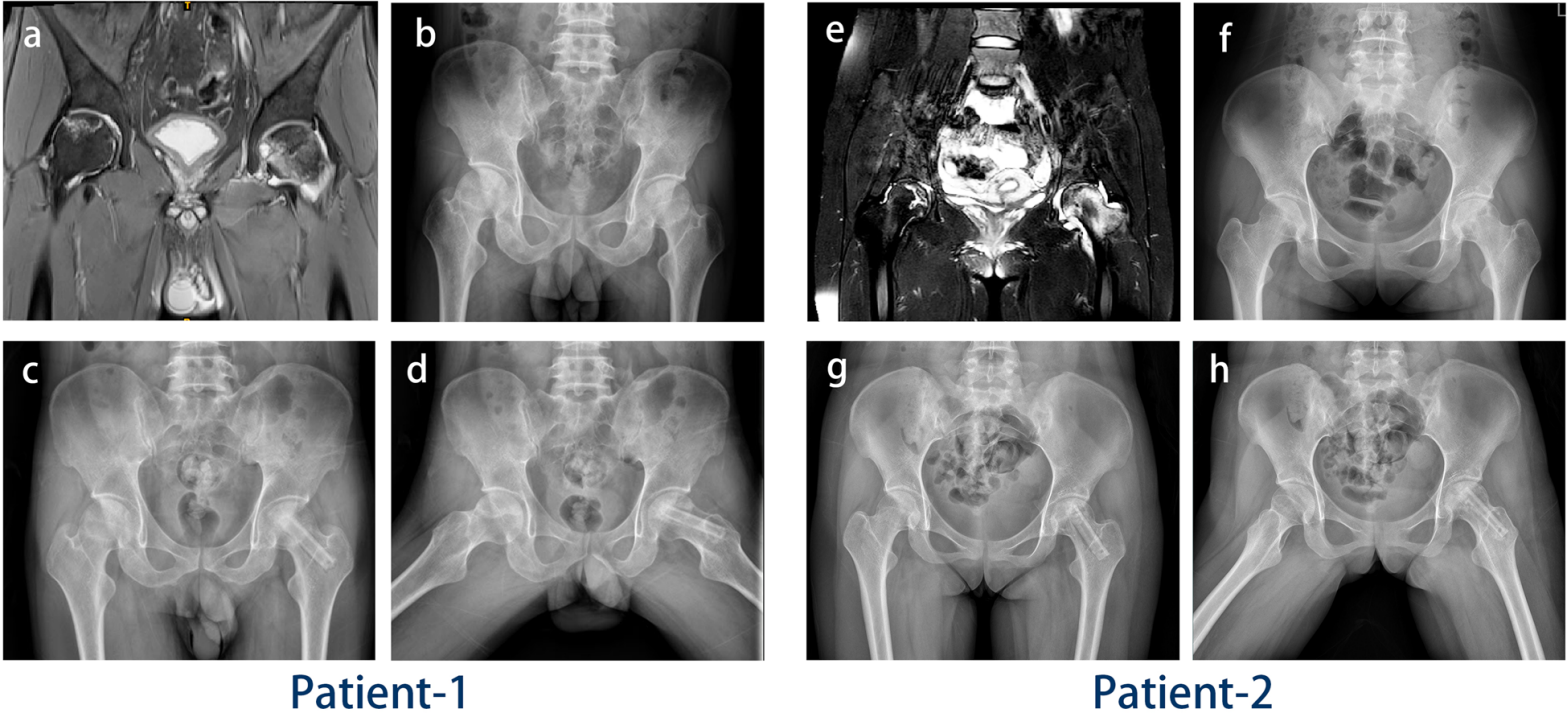
Two patients’ pre- and postoperative images. **a**–**d** Patient 1,the case in the left hip was classified as JIC C1 NONFH. **e**–**h** Patient 2, the case in the left hip was classified as JIC C2 NONFH. The top left panels (**a**, **e**) show the preoperative T2-weighted MRI scan. The top right panels (**b**, **f**) show the preoperative X-ray. The bottom panels show the postoperative X-ray, including patient 1’s left hip after the LBT at the 4-week follow-up (**c**, **d**) and patient 2’s left hip after the LBT at the 4-week follow-up (**g**, **h**).

To verify the finite element models, two patients diagnosed with bilateral NONFH and treated at our hospital were enrolled (Table 2), and the cases were classified as type C1 and C2 according to the JIC classification system (both in stage 2, Fig. 3a, e); the same FEA method was used to compare the average stress at different regions before and after surgery with the LBT. The preoperative and 4-week postoperative CT scans were used for analysis.

**Table 2:** Patient parameter

Parameter	Patient 1	Patient 2
Sex	Male	Female
Age	38	21
Height (cm)	173	160
Weight (kg)	65	50
BMI	21.7	19.5
Bilateral	yes	yes
Surgical hip	Left	Left
JIC classification	Type C1	Type C2
Length of grafted fibular (cm)	7.94	7.13
FEA	Pre-/postoperation	
Element	265388/263941	235848/251905
Node	54869/54768	49077/52590

## Results

**Fig. 4:**
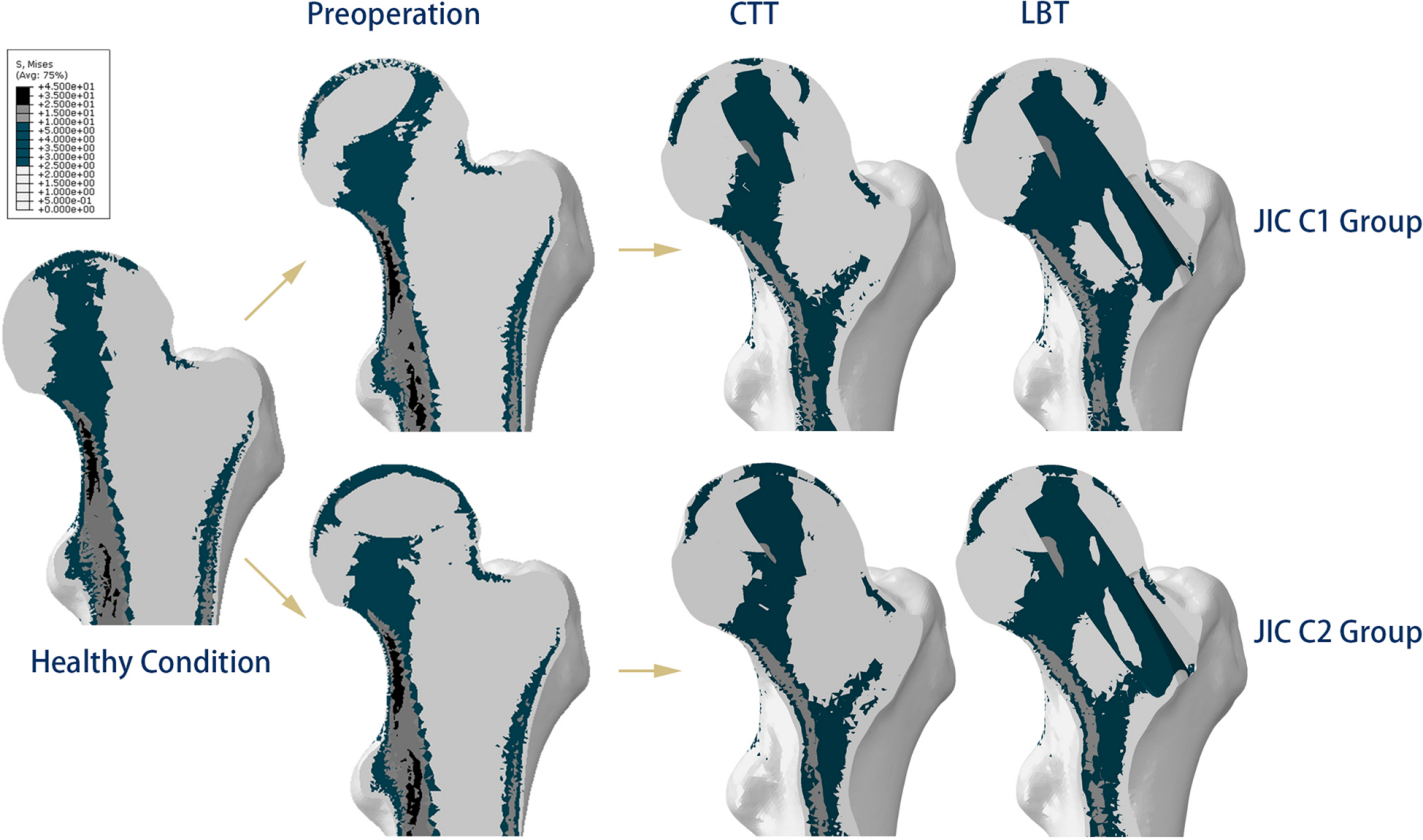
The stress distributions in the finite element models. The stress transfer path is shown in the healthy subject-derived models. The preoperative models for JIC C1 and JIC C2. The postoperative models for the CTT and LBT are displayed by the necrotic type

**Fig. 5:**
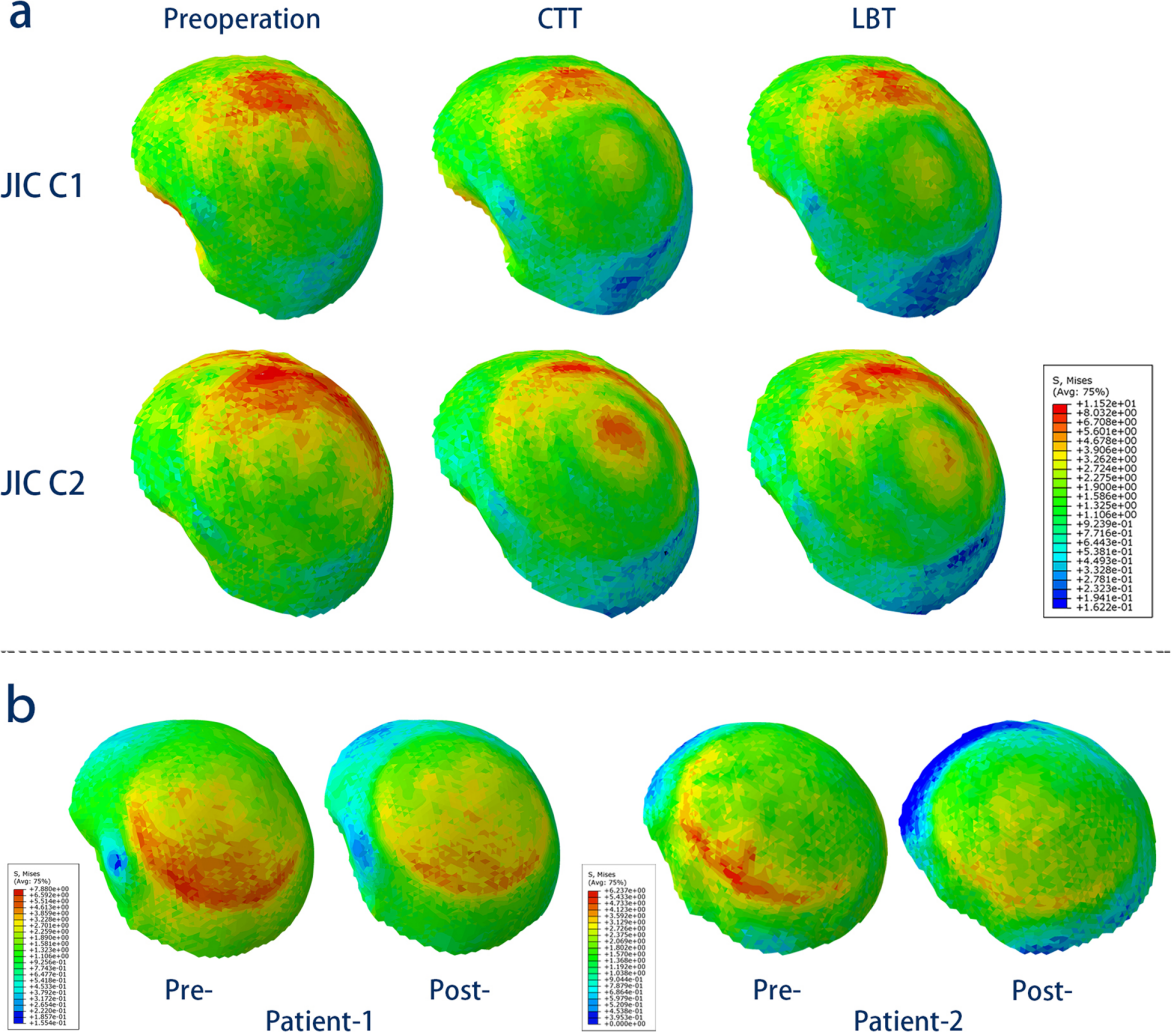
Stress distributions on the femoral head surface. **a** The healthy subject-derived models. **b** Two patient-specific models

As shown in Fig. 4, in the simulation of maximal walking impact, relatively high stresses appeared mainly from the weight-bearing area to the calcar in the healthy model, forming the normal stress transfer path of the proximal femur. However, the stress transfer path was blocked in the necrotic models. After the CTT/LBT was performed, the implanted fibular graft partially bore the high stresses and contributed to the reconstruction of the stress transfer path. The distributions of stress on the femoral head surfaces in the healthy-derived models and patient-specific models are demonstrated in Fig. 5.

**Fig. 6:**
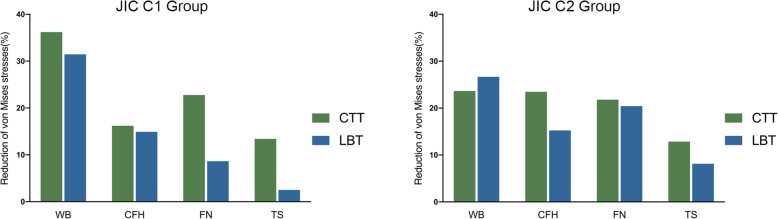
The reduction in the average von Mises stresses following the CTT/LBT in the JIC C1 and C2 groups. CTT, core track technique; LBT, light bulb technique; WB, weight-bearing area; CFH, central femoral head; FH, femoral neck; TS, trochanteric and subtrochanteric region.

In all the postoperative simulation models, the average von Mises stress decreased in all four regions (Fig. 6 and Table 3), and the largest magnitude of reduction occurred in the weight-bearing area (23.63–36.19%). Among the techniques, the CTT yielded a larger reduction than did the LBT in the JIC C1 group (36.19% vs 31.45%), whereas the LBT yielded a larger reduction than did the CTT in the JIC C2 group (26.76% vs 23.63%). In other regions, the CTT led to a larger reduction of stresses in both the JIC C1 and JIC C2 groups than did the LBT (range of reduction, 12.86–23.48% vs 2.51–20.43%). The stiffness increased in both the CTT and LBT groups (2.26–9.40%), and the magnitude of increase for the CTT was larger than that for the LBT for both types of necrosis (Table 3). No yielding units appeared postoperatively in the proximal femur.

**Table 3:** The average von Mises stress and stiffness in each region and reduced percentage in CTT and LBT

	Stress (Mpa)				Stiffness (*N*/mm)
	WB	CFH	FN	TS	
JIC C1					
Preoperation	3.63	2.3	4.85	2.36	3068.48
Post-CTT	2.32 (− 36.19%)	1.93 (− 16.19%)	3.75 (− 22.76%)	2.04 (− 13.41%)	3356.89 (+ 9.40%)
Post-LBT	2.49 (− 31.45%)	1.95 (− 14.91%)	4.43 (− 8.65%)	2.30 (− 2.51%)	3227.45 (+ 5.18%)
JIC C2					
Preoperation	3.39	2.35	5.16	2.32	3205.67
Post-CTT	2.59 (− 23.63%)	1.80 (− 23.48%)	4.04 (− 21.78%)	2.02 (− 12.86%)	3454.55 (+ 7.76%)
Post-LBT	2.49 (− 26.76%)	1.99 (− 15.24%)	4.11 (− 20.43%)	2.13 (− 8.14%)	3278.12 (+ 2.26%)

*WB* weight-bearing area, *CFH* central femoral head, *FH* femoral neck, *TS* trochanteric and subtrochanteric region. The value in parentheses is the relative preoperative percentage

The simulation of the patient-specific models revealed that both the average von Mises stresses and stiffness decreased after the LBT, which was consistent with the results of the healthy subject-derived models (Table 4). Compared with patient 1 (JIC type C1), patient 2 (JIC type C2) demonstrated larger changes in the abovementioned parameters. Postoperative X-ray radiography demonstrated that the femoral heads retained their shape (Fig. 3), and neither patient complained of hip pain at the 1-year follow-up.

**Table 4:** The average von Mises stress and stiffness of patient-specific models

	Stress (Mpa)				Stiffness (N/mm)
	WB	CFH	FN	TS	
Patient 1					
Preoperation	2.60	1.97	10.85	6.62	555.94
Post-LBT	2.54 (− 2.48%)	1.70 (− 13.62%)	9.36 (− 13.76%)	5.84 (− 11.82%)	672.55 (+ 20.98%)
Patient 2					
Preoperation	2.17	2.09	11.91	7.67	433.43
Post-LBT	2.04 (− 6.00%)	1.57 (− 24.90%)	9.6 (− 19.40%)	6.16 (− 19.69%)	555.8 (+ 28.23%)

*WB* weight-bearing area, *CFH* central femoral head, *FH* femoral neck, *TS* trochanteric and subtrochanteric region. The value in parentheses is the relative preoperative percentage

## Discussion

Researchers have tried to use finite element analysis to investigate the biomechanical effectiveness of core decompression [24], rod implantation [27, 28], and the CTT [19] in preserving the hip joint in NONFH surgeries. However, there have been no such investigations on the LBT. Although the LBT has demonstrated a relatively higher success rate in the treatment of NONFH [10], there are still concerns about the associated risk of fractures, and few studies have compared the LBT with other techniques [8, 14]. In this study, we performed finite element analysis to compare the LBT with the CTT and provide biomechanical references for the current debate. The biomechanical aspects of both the LBT and CTT were assessed with respect to the JIC types.

In terms of the validation of finite elemental models in this study, we compared our results with references and the physical characteristics of the hip. Moreover, one patient with type C1 and one patient with type C2 were assessed to validate the biomechanical properties of the LBT. The shape and location of the stress transfer path in this study are consistent with those in previous studies, which showed that the stress transfer path is consistent with the distribution of bone density [19, 29, 30]. In addition, the average stress of the weight-bearing area after fibula implantation ranged from 2.32 to 2.59 Mpa, which was in accordance with that in a recent study [28]. Therefore, the simulation results could reflect the physical status of the hip and could be used to analyse the effects of the LBT and CTT.

The necrosis of the femoral head varies from case to case; thus, appropriate classification is a prerequisite for modelling. The JIC classification directly reflects the relative position between the necrotic lesions and weight-bearing area [31–33] and has been proven to be an appropriate classification system for predicting the stress distribution of the hip joint in vivo [19, 30, 34, 35]. Regarding the JIC classification system, type C1 and C2 are recommended to undergo joint-preserving therapies, so both types were included in this study. Kuroda et al. [16] reported that the JIC classification system can assist with the selection of therapeutic options before the collapse of the femoral head, particularly for patients with JIC type C2 NONFH [35]. As type C2 cases account for 53% of all cases of NONFH [36] and have been recently reported to be associated with a high collapse rate [35], treatment for this type should be emphasized.

In all postoperative finite element models, the average von Mises stresses were alleviated in the studied regions after both the LBT and CTT. It is reasonable that the former necrotic areas were replaced by fibular grafts. Additionally, the stiffness increased in all models (Table 3), which indicated that both techniques can effectively improve the structural stability of the proximal femur and prevent the collapse of the femoral head. These results were supported by the simulation of patient-specific models (Table 4 and Fig. 6). The stiffness after the CTT was 4.01–5.38% higher than that after the LBT, which might be related to the differences in the drilling method and the length of grafted fibula. Note that the CTT needed a longer grafted fibula (Table 1).

The average stress of the weight-bearing area, which reflects its collapse risk, is the most important measure in this study. Interestingly, the two JIC groups showed opposite results (Table 3). In the JIC C1 group, the average stress in the weight-bearing area of the femoral head after the CTT was relatively lower, whereas it was relatively lower after the LBT in the JIC C2 group. The inconsistency in results may have been caused by the combination of several factors. First, the tail of the grafted fibula was partially supported by the lateral cortex of the femur in the LBT, while there was no such support by the cortex in the CTT. Second, in the JIC C2 group, the angle formed between the grafted fibula and the stress transfer path was smaller than that in the JIC C1 group, and the force transmitted to the fibula was larger, so the mechanical effect of the LBT was more favourable than that of the CTT in the JIC C2 group. Moreover, type C2 cases account for the highest proportion (53%) of all NONFH cases, meaning that more patients who underwent the LBT received the appropriate treatment. This may be the reason why the clinical success rate was relatively higher for the LBT than for that CTT (94.6–96% vs 60–90%).

Regarding FVFG procedures, surgeons are concerned that a structural defect will form at the proximal femur, which can cause the femoral neck and subtrochanteric regions to fracture [12, 37]. Aldridge and Urbaniak [15] suggested that the LBT raises the stress by opening a window in the femoral neck. However, our results showed that the average stresses of the femoral neck, trochanteric, and subtrochanteric regions were lower after the LBT than they were preoperatively. Additionally, no yielding units appeared in these regions, meaning that the risk of fracture in these regions decreased with the LBT. Gao et al. [10] reported 578 hips that underwent the LBT procedure did not develop proximal femoral fractures postoperatively. However, as a comparative technique in this study, the CTT was reported to have a 0.7–1% fracture rate in previous studies [11, 15, 37], and all of those fractures occurred in the intertrochanteric and subtrochanteric regions after a fall [37]. Since the integrity of the lateral femoral cortex is crucial for the structural strength and in preventing intertrochanteric and subtrochanteric fractures [38, 39], the impact of the CTT on this integrity needs to be studied further. From our clinical experience, adequate postoperative rehabilitation and fall prevention may reduce the prevalence of fractures.

The limitations of this study should be clarified. First, only one healthy hip joint model was used for simulation, and two patient-specific models were used for validation. However, all comparisons were based on the healthy hip joint model, so the risk of deviation caused by the differences in the models was eliminated. Second, our study used a simplified model, so some details of biological models were ignored. For instance, we did not consider internal fixation for both the LBT and CTT; instead, we considered that bony union was achieved. However, our study focused on the structural changes in the proximal femur, similar to previous studies [19, 40], and the simplification likely did not affect the results. Last but not least, multiple factors affecting graft survival were not taken into consideration, such as the accuracy of surgery, the occurrence of revascularization, and an imbalance in creeping substitution [41–43]. Additional studies need to be conducted to clinically compare the LBT and CTT on the basis of these factors and biomechanical outcomes.

## Conclusion

The biomechanical performance of the LBT and CTT differs by the JIC type of NONFH. In terms of preventing the collapse of the femoral head, the LBT may be more effective for JIC C2 NONFH and can be chosen as an alternative, while the CTT is still a better option for JIC type C1 NONFH. The biomechanical properties of NONFH can be improved by both techniques, with the patients’ proximal femur stress being reduced and the structural stiffness being increased.

## Supplementary information

**Additional file 1.** Supplementary materials.

## Data Availability

All of the data is available in contact with the corresponding author.

## Abbreviations

FVFG: Free vascularized fibula graft
NONFH: Nontraumatic osteonecrosis of femoral head
LBT: Light bulb technique
CTT: Core track technique
FEA: Finite element analysis
JIC: Japanese Investigation Committee
