# Supplementary material for: Biomechanical analysis of fibular graft techniques for nontraumatic osteonecrosis of the femoral head: a finite element analysis
Source: J Orthop Surg Res. 2020 Aug 17;15:335. doi: 10.1186/s13018-020-01867-4 (PMC7433362; doi:10.1186/s13018-020-01867-4)

**Supplementary materials**

In addition, two patients diagnosed with bilateral NONFH and treated at our hospital were enrolled (Table 1); the cases were classified as Type C1 and C2 according to the JIC classification system (both in stage 2, Fig. 1a, e); the same FEA method was used to compare the average stress of the different regions before and after surgery with the LBT. The preoperative and 4-week postoperative CT scans were used for analysis.

The simulation of patient-specific models revealed that both the average von Mises stresses and stiffness decreased after the LBT, which was consistent with the results of the healthy subject-derived models (Table 2 & Fig. 1 i, j). Postoperative X-ray radiography demonstrated that the femoral heads retained their shape (Fig. 1), and neither patient complained of hip pain at the 1-year follow-up.

**Table**

Table 1 Patient parameter

| Parameter | Paitient-3 | Paitient-4 |
| --- | --- | --- |
| Sex | Female | Male |
| Age | 38 | 23 |
| Height (cm) | 155 | 172 |
| Weight(Kg) | 55 | 60 |
| BMI | 22.8 | 20.3 |
| Bilateral | yes | yes |
| Surgical hip | Right | Left |
| JIC Classification | Type C1 | Type C2 |
| Length of grafted fibular (cm) | 6.67 | 6.25 |
| FEA | Pre/post operation | |
| Element | 250814/257049 | 258921/257657 |
| Node | 52100/53402 | 53643/53398 |

Table 2 The average Von Mises stress and stiffness of patient-specific models

|  |  | Stress (Mpa) | | | | Stiffness |
| --- | --- | --- | --- | --- | --- | --- |
|  |  | WB | CFH | FN | TS | (N/mm) |
| Patient-3 | |  |  |  |  |  |
|  | preoperation | 1.87 | 1.65 | 9.73 | 5.96 | 596.01 |
|  | post-LBT | 1.79 (-4.28%) | 1.51 (-8.48%) | 9.37 (-3.70%) | 5.68 (-4.70%) | 600.7 (+0.79%) |
| Patient-4 | |  |  |  |  |  |
|  | preoperation | 2.61 | 1.92 | 8.49 | 5.01 | 612.49 |
|  | post-LBT | 2.26 (-13.51%) | 1.44 (-24.90%) | 8.06 (-5.09%) | 4.87 (-2.92%) | 851.31 (+38.99%) |
| WB: weight-bearing area; CFH: central femoral head; FH: femoral neck; TS: trochanteric and subtrochanteric region; The value in parentheses is the relative preoperative percentage. | | | | | | |

**Figure**


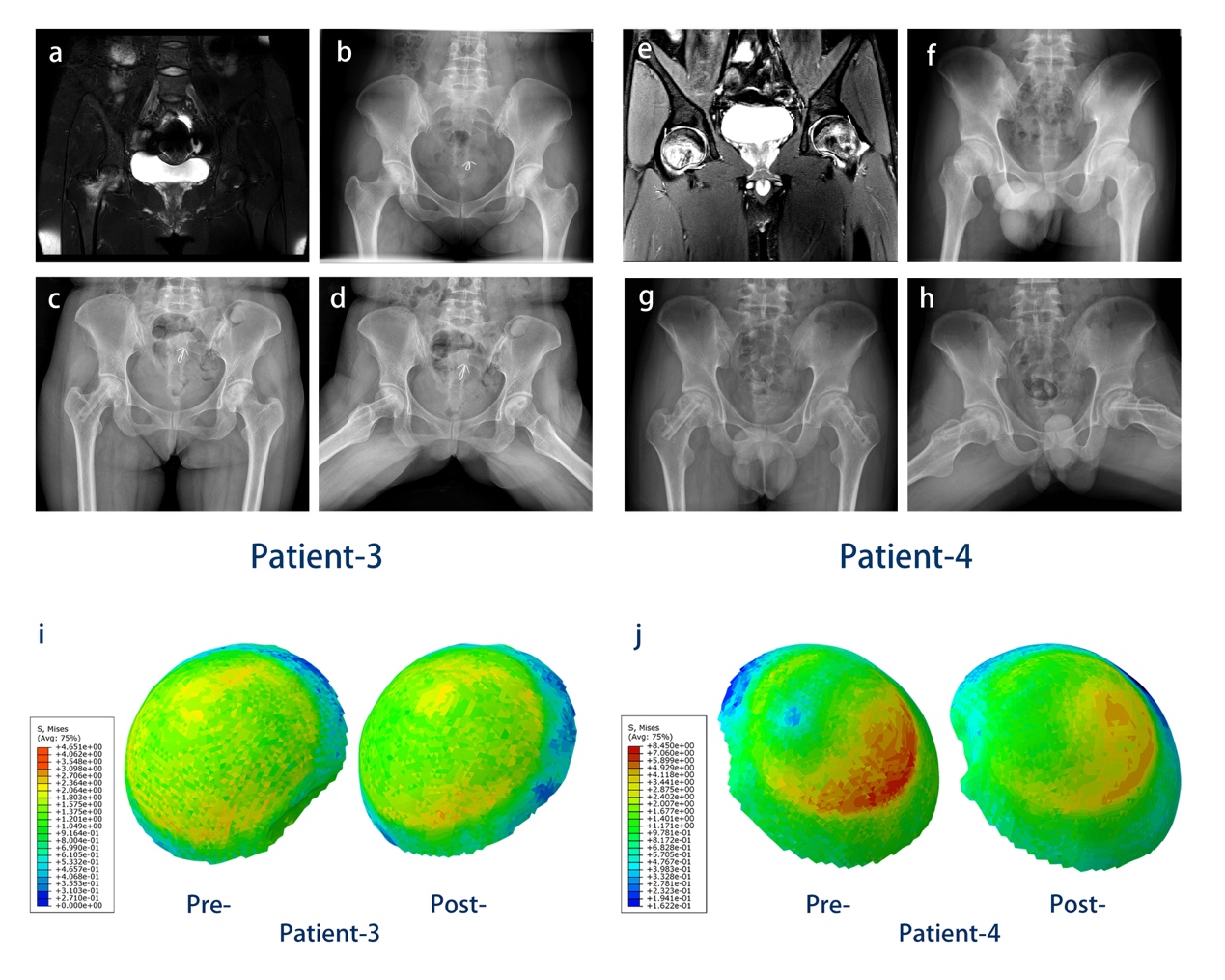


**Fig. 1 Two patients’ pre- and postoperative images and stress distributions.** (a-d) Patient-3, the case in the right hip was classified as JIC C1 NONFH; (e-h) Patient-4, the case in the left hip was classified as JIC C2 NONFH. The top left panels (a, e) show the preoperative T2-weighted MRI; the top right panels (b, f) show the preoperative X-ray; the bottom panels show the postoperative X-ray, including patient-3’s right hip after the LBT at the 6-month follow-up (c, d), and patient-4’s left hip after the LBT at the 6-month follow-up (g, h). (i-j)The stress distributions on the femoral head surface.

**English editing certificate**


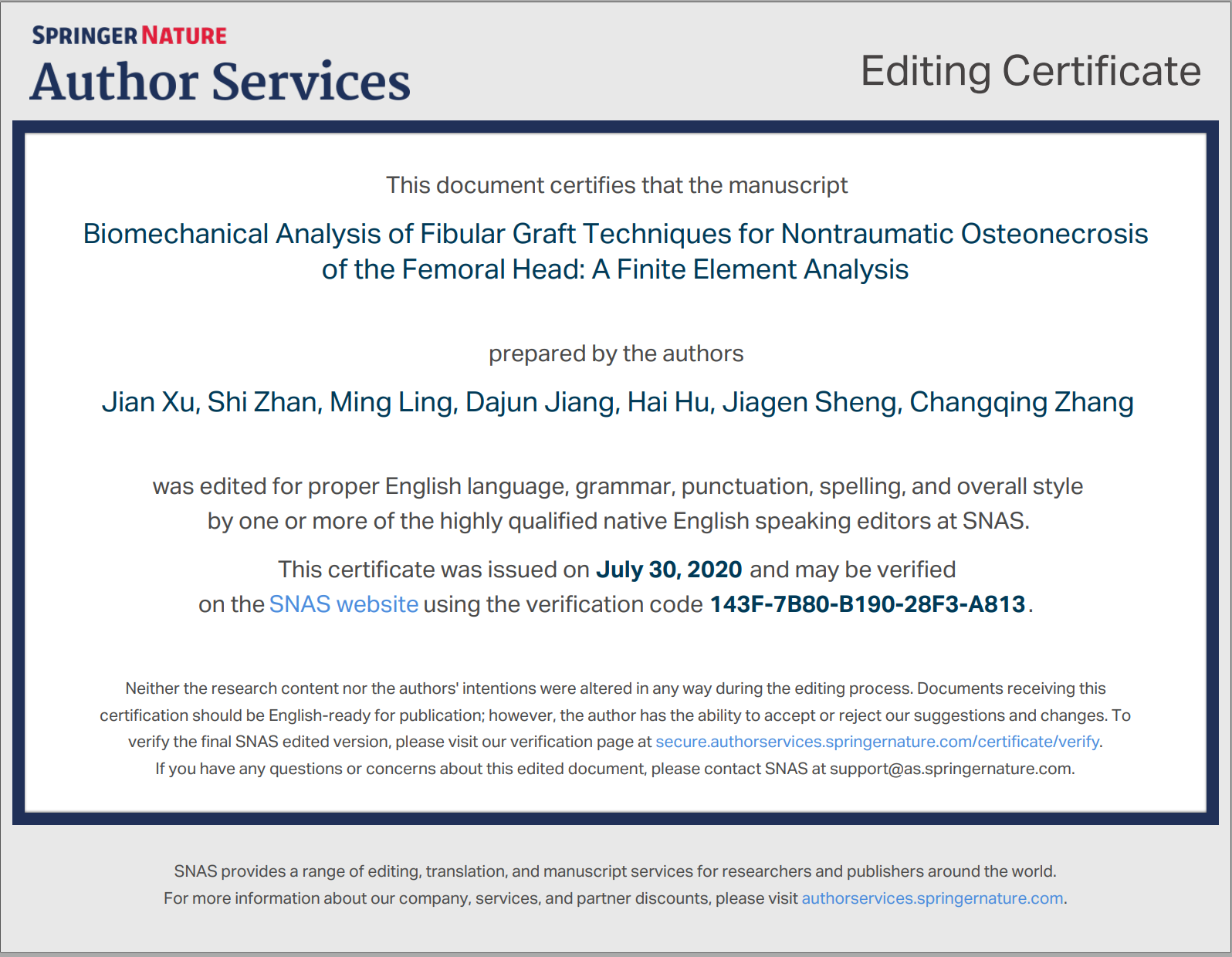

Supplement: Supplementary file 1 — Additional file 1. Supplementary materials. [file 13018_2020_1867_MOESM1_ESM.docx]
